# Reticulocalbin-1 Facilitates Microglial Phagocytosis

**DOI:** 10.1371/journal.pone.0126993

**Published:** 2015-05-18

**Authors:** Ying Ding, Nora B. Caberoy, Feiye Guo, Michelle E. LeBlanc, Chenming Zhang, Weiwen Wang, Feng Wang, Rui Chen, Wei Li

**Affiliations:** 1 Bascom Palmer Eye Institute, Dept. of Ophthalmology, University of Miami School of Medicine, Miami, FL, 33136, United States of America; 2 Neuroscience Program, University of Miami School of Medicine, Miami, FL, 33136, United States of America; 3 School of Life Sciences, University of Nevada Las Vegas, Las Vegas, NV, 89154, United States of America; 4 Jinan 2^nd^ People’s Hospital, Jinan, 250001, China; 5 Dept. of Molecular & Human Genetics, Baylor College of Medicine, Houston, TX, 77030, United States of America; National Institute of Biological Sciences, Beijing, CHINA

## Abstract

Phagocytosis is critical to the clearance of apoptotic cells, cellular debris and deleterious metabolic products for tissue homeostasis. Phagocytosis ligands directly recognizing deleterious cargos are the key to defining the functional roles of phagocytes, but are traditionally identified on a case-by-case basis with technical challenges. As a result, extrinsic regulation of phagocytosis is poorly defined. Here we demonstrate that microglial phagocytosis ligands can be systematically identified by a new approach of functional screening. One of the identified ligands is reticulocalbin-1 (Rcn1), which was originally reported as a Ca^2+^-binding protein with a strict expression in the endoplasmic reticulum. Our results showed that Rcn1 can be secreted from healthy cells and that secreted Rcn1 selectively bound to the surface of apoptotic neurons, but not healthy neurons. Independent characterization revealed that Rcn1 stimulated microglial phagocytosis of apoptotic but not healthy neurons. Ingested apoptotic cells were targeted to phagosomes and co-localized with phagosome marker Rab7. These data suggest that Rcn1 is a genuine phagocytosis ligand. The new approach described in this study will enable systematic identification of microglial phagocytosis ligands with broad applicability to many other phagocytes.

## Introduction

Phagocytosis of apoptotic neurons, cellular debris and deleterious metabolic products, also called efferocytosis, is pivotal to maintain tissue homeostasis, prevent autoimmune response and resolve inflammation [1,2]. For example, the importance of microglial phagocytosis is highlighted by phagocytic receptor TREM2, whose mutations cause microglial dysfunction, neuroinflammation and neurodegenerative diseases, such as Alzheimer’s disease and Nasu-Hakola disease [3,4]. Microglial dysfunction in aged brain has been implicated in age-dependent neurodegenerations [5,6]. Phagocytosis ligands directly recognize deleterious cargos, bridge them to microglia and initiate cargo engulfment by activating cognate phagocytic receptors. In this regard, these ligands are the key to defining the physiological and pathological roles of microglial phagocytosis. Like other cellular ligands, however, phagocytosis ligands are traditionally identified in individual cases with technical challenges. As a result, only a limited number of microglial ligands have been reported [1]. Most of them were originally identified in bone marrow-derived macrophages and extrapolated to yolk sac-derived microglia [1,7]. In fact, we really do not know if such extrapolations can be broadly applied to microglia, how many unknown ligand-receptor pathways are yet to be identified and which ones may be relatively active, disease-related or age-dependent. For instance, despite identification of TREM2 as a phagocytic receptor 14 years ago [8], its ligand(s) remains elusive [9]. It is even more daunting to identify disease-related TREM2 ligands for understanding the pathological roles of microglial phagocytosis. Moreover, no single age-dependent phagocytosis pathway or signaling molecule has been identified.

Rcn1 belongs to the family of CREC (Cab45, reticulocalbin, ERC-55 and calumenin) proteins that were characterized as Ca^2+^-binding proteins in the endoplasmic reticulum (ER) with EF hands [10,11]. Rcn1 is widely expressed in various fetal and adult organs, including the CNS [12]. In fetal brain, Rcn1 was found in ependymal cells, neuroblasts and a minority of glial cells. In adult brain and spinal cord, Rcn1 was detected in all neurons except Purkinje cells. Activated astrocytes in various conditions showed strong staining of Rcn1. Despite its extensive expression, the functional roles of Rcn1 remain unknown.

Here we identified Rcn1 as a microglial phagocytosis ligand by a new functional screening approach. Independent characterization showed that Rcn1 extrinsically promoted microglial phagocytosis of apoptotic but not healthy neurons. Apoptotic neurons engulfed through Rcn1-mediated pathway were targeted to phagosomes and co-localized with a phagosome marker. Rcn1 was secreted into culture medium and preferentially bound to apoptotic but not healthy neurons. These data suggest that Rcn1 is a genuine microglial phagocytosis ligand. Furthermore, the new approach described in this study will enable systematic delineation of microglial phagocytosis ligands.

## Materials and Methods

### Cell culture

BV-2 microglial, J774 macrophage and Neuro-2A cell lines were previously described [13]. HEK293 cell was purchased from ATCC. All cells were cultured in Dulbecco's modified essential medium supplemented with 10% FBS and 2 mM L-glutamine.

### Identification of phagocytosis ligands

Open reading frame phage display (OPD) cDNA library generated from mouse embryos at E18 was described previously [14]. Phagocytosis-based functional cloning (PFC) selection with BV-2 microglial cells was carried out as described [15]. Briefly, the library was amplified in BLT5615 bacteria, precipitated with polyethylene glycol, resuspended in the complete medium and incubated with BV-2 cells at 4°C for 30 min. After washing, cells with bound phages were incubated at 37°C for 30 min for phagocytosis. Surface-bound unphagocytosed phages were removed with low pH isotonic stripping buffer [13]. Phagocytosed clones were released by cell lysis, amplified and used as input for the next round of OPD/PFC selection. After 3 rounds of selection, the cDNA inserts of enriched clones were amplified by PCR with primer 5’-NNNNNNCCGGGCAGCGGTTCAGGCTC-3’ and 5’-NNNNNNCCGCTCCCACTACCCTCGAG-3’, and identified by next generation DNA sequencing (NGS).

### Plasmids

A cDNA clone of Rcn1 (GenBank accession #BC049108) was obtained from Open Biosystems. The internal Not I site in the coding region was destroyed by a silent mutation. The full-length coding sequence with a C-terminal FLAG tag was amplified by PCR and cloned into pEGFP-N1 plasmid (Clontech) to replace EGFP at Kpn I and Not I sites and yield Rcn1-FLAG plasmid. A similar Rcn1-FLAG plasmid was constructed without the signal peptide. In addition, the coding sequence was amplified by PCR, digested with Bgl II and EcoR I, and cloned into pGEX-2T plasmid (GE Healthcare) at BamH I and EcoR I sites to yield a plasmid encoding glutathione S-transferase-Rcn1 fusion protein (GST-Rcn1). The resulting plasmids were verified by sequencing. GFP-FLAG and FLAG-Tulp1 plasmids were described previously [15].

### Phage construction

The coding sequence of Rcn1 was amplified by PCR and cloned into T7Bio3C phage vector [16] at Not I and Xho I sites to generate Rcn1-Phage. Clonal phage displaying green fluorescent protein (GFP-Phage) was constructed in the same manner. Clonal phages were verified by sequencing.

### Phage phagocytosis assay

Rcn1-Phage, GFP-Phage and Control-Phage (i.e., T7Bio3C phage vector without foreign cDNA insert) were amplified, precipitated and used for OPD/PFC selection in 12-well plates as above. After binding, washing, phagocytosis and stripping, internalized phages were released by cell lysis and quantified by plaque assay as plaque forming units (pfu)/well [13].

### Protein purification

GST-Rcn1 and control GST were expressed in BL21(DE3) and purified using glutathione columns, as described [16]. Purified proteins were dialyzed and analyzed by SDS-PAGE.

### Phagocytosis assay

BV-2 microglia or J774 macrophages were seeded on coverslips precoated with poly-L-lysine in 12-well plates, and cultured to ~80% confluence. Neuro-2A cells were transfected with Rcn1-FLAG, FLAG-Tulp1 or GFP-FLAG plasmid by jetPRIME reagents (Polyplus), cultured for 48 h, and then treated with or without etoposide (200 nM) for 16 h to induce apoptosis. Cells were collected and labeled with pHrodo succinimidyl ester (Life Technologies) [17]. Briefly, apoptotic or healthy cells (1 x 10^6^ cells/ml) were incubated with pHrodo (20 ng/ml) for 15 min at 37°C. After washing with PBS containing 1% bovine serum albumin (BSA), labeled cells (3 x 10^5^ cells/well) were incubated with the phagocytes for 3 h. Alternatively, BV-2 cells were transfected with Rcn1-FLAG or control plasmid for 48 h and then incubated with pHrodo-labeled cells for phagocytosis assay. The phagocytes were washed, fixed with 4% buffered paraformaldehyde and analyzed by confocal microscopy to detect ingested cargos [15,18]. The percentage of microglia with phagocytosed cargos were quantified by ImageJ software (NIH).

Alternatively, Neuro-2A cells were treated with or without etoposide to induce apoptosis, incubated with BV-2 cells in the presence of purified GST-Rcn1 or GST for phagocytosis, and analyzed by confocal microscopy as above.

In addition, FITC latex beads (2 μm in diameter, Sigma) were incubated with BV-2 cells in the presence of GST-Rcn1 or GST for phagocytosis and analyzed as above.

### Immunocytochemistry

Microglia with phagocytosed pHrodo-labeled cargos were fixed, permeabilized with PBS containing 1% Triton X-100, and immunostained with anti-Rab7 antibody (Abcam) and Alexa Fluor 488-labeled secondary antibody. The nuclei were visualized by DAPI. The intracellular fluorescence signals were analyzed by confocal microscopy.

### Rcn1 extracellular trafficking and binding to apoptotic cells

Neuro-2A cells were transfected with Rcn1-FLAG plasmid and treated with or without etoposide as above. Rcn1-FLAG in the conditioned medium of apoptotic and healthy cells was pulled down by anti-FLAG M2 monoclonal antibody (mAb) (Sigma) and protein A beads, and analyzed by Western blot using anti-FLAG mAb [18]. Furthermore, Rcn1-FLAG on the surface of apoptotic or healthy cells expressing Rcn1-FLAG was detected by FITC-labeled anti-FLAG mAb without permeabilization and analyzed by fluorescence microscopy. Apoptotic cells were labeled with propidium iodide.

### Flow cytometry

HEK293T cells were transfected with Rcn1-FLAG, FLAG-Tulp1 or GFP-FLAG plasmid. After 48 h, lysates were prepared from the cells without any detergent by 3 cycles of freeze-thaw, followed by centrifugation at 16,000 x g for 20 min and filtration through a 0.2-μm filter. Neuro-2A cells were treated with or without etoposide to induce apoptosis and incubated with the lysates at 4°C for 1 h with rotation. Cell surface-bound FLAG-tagged proteins were detected by FITC anti-FLAG mAb and analyzed by flow cytometry. Apoptotic cells were labeled with propidium iodide. Additionally, BV-2 cells were incubated with GST-Rcn1 or GST control, washed and analyzed by flow cytometry using FITC-anti-GST antibody (AnaSpec). FITC and propidium iodide were excited 488 and 561 nm, and detected at 525 and 612 nm, respectively.

### Statistical analysis

Data were expressed as means + s.e.m. and analyzed by unpaired Student’s t-test. Data were considered significant when *P* < 0.05.

## Results

### Identification of Rcn1 as a phagocytosis ligand

**Fig 1:**
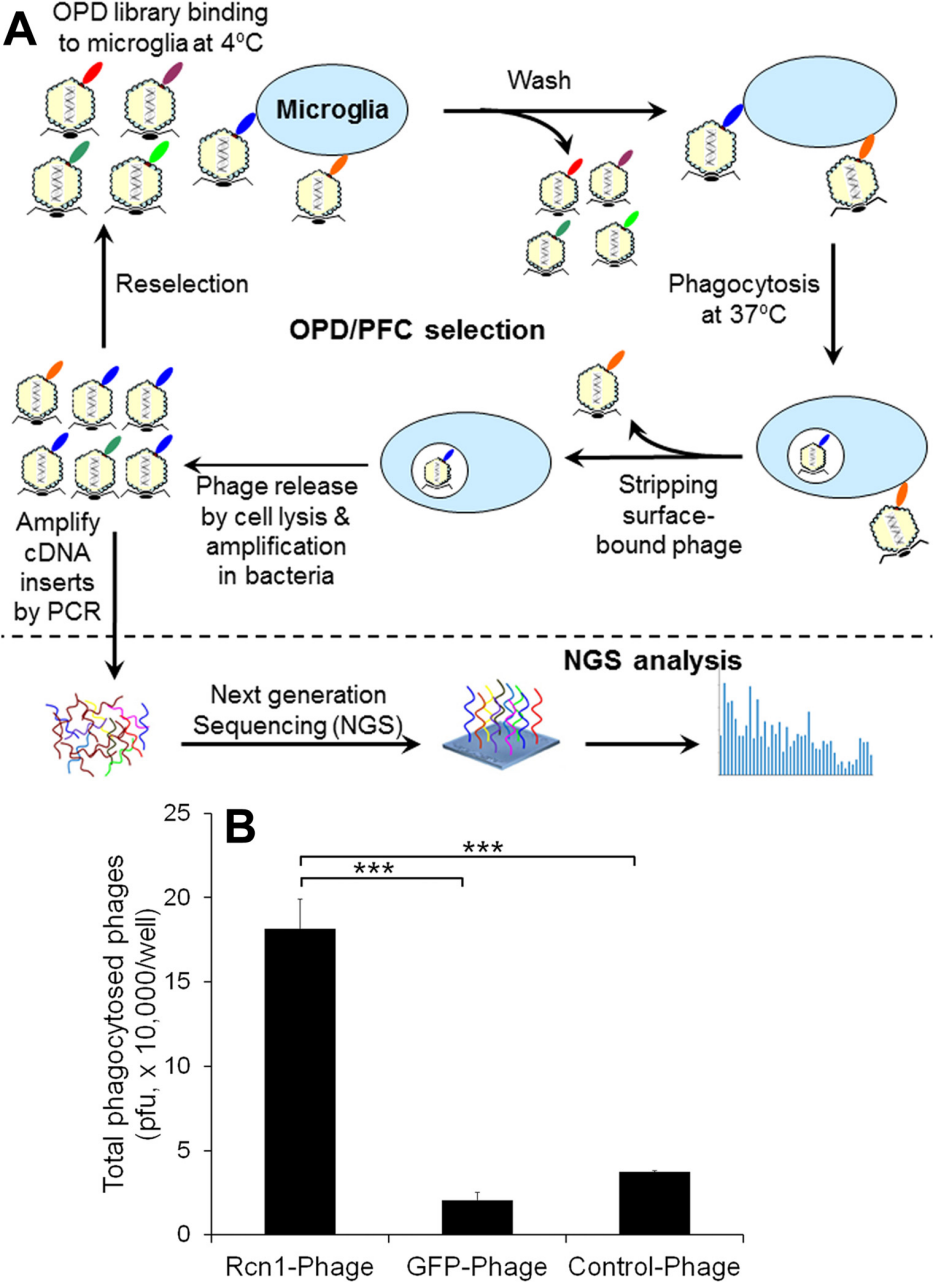
Identification of Rcn1 as a microglial phagocytosis ligand. (A) Schematics of OPD-NGS. OPD/PFC selection was performed by incubating OPD cDNA library with BV-2 microglial cells at 4°C. After washing, cells were incubated at 37°C for phagocytosis. Surface-bound unphagocytosed phages were removed by stripping with low pH isotonic buffer. Phagocytosed phages were released by cell lysis, amplified and used as input for the next round of selection. After 3 rounds of selection, the cDNA inserts of enriched clones were amplified by PCR and identified by NGS. (B) Verification of Rcn1-Phage phagocytosis by microglia. Clonal Rcn1-Phage, GFP-Phage or Control-Phage was incubated with BV-2 cells at 4°C. After washing, cells were incubated at 37°C to allow bound phages to be phagocytosed. After removal of surface-bound unphagocytosed phage, internalized phages were released by cell lysis and quantified by plaque assay (+ s.e.m., *** *P*<0.0001, n = 4, t-test).

Phagocytosis ligands are traditionally identified on a case-by-case basis with technical challenges. To tackle the challenges, we recently developed OPD and PFC for unbiased identification of phagocytosis ligands in the absence of receptor information [15,16,19]. The validity of OPD/PFC has been demonstrated by identifying and independently characterizing phagocytosis ligands for retinal pigment epithelial (RPE) cells [15,18]. Here we applied this new approach to microglial phagocytosis by performing 3 rounds of OPD/PFC selection with BV-2 microglial cells as in Fig 1A. Instead of performing labor-intensive manual screening for positive clones with increased internalization activity [15], we analyzed the cDNA inserts of enriched clones by NGS and identified a total of 2,241,076 valid sequence reads. All identified sequences were aligned against NCBI CCDS database to identify ligands with internalization activity. We list top known or putative phagocytosis ligands in Table 1, including Gas6 and Tulp1 [18,20]. One of the identified proteins with a high copy number of cDNA was Rcn1.

**Table 1:** Partial list of known or putative phagocytosis ligands identified by OPD-NGS.

CCDS_ID	Protein	Copy #	Rank	Comments and references
CCDS40232	Gas6	495,647	1	Known ligand for Tyro3, Axl and Mer receptors [20].
CCDS28578	Tulp1	149,677	4	Known ligand for Tyro3, Axl and Mer receptors [18].
CCDS28713	Abcf1	28,336	11	Verified as RPE phagocytosis ligand (unpublished data, Guo et al.) [15].
CCDS16498	Rcn1	10,221	31	Verified as microglial phagocytosis ligand in this study.
CCDS19483	Sparcl1	3,332	72	Reported as a phagocytosis-promoting gene [43].
CCDS38035	Eif3a	2,990	77	Eif3a was reported as a phagocytosis-promoting gene [44].
CCDS28285	App	2,167	99	Amyloid β peptides derived from App is cleared by microglia [1,37].

To verify the finding, we constructed clonal phage Rcn1-Phage to display Rcn1 and analyzed its internalization activity in BV-2 cells. GFP-Phage and Control-Phage were used as negative controls. The results showed that Rcn1-Phage was phagocytosed by BV-2 cells at a significantly higher activity than GFP-Phage and Control-Phage (Fig 1B), suggesting that Rcn1 is a candidate ligand for microglial phagocytosis.

### Rcn1 stimulates BV-2 microglial phagocytosis

**Fig 2:**
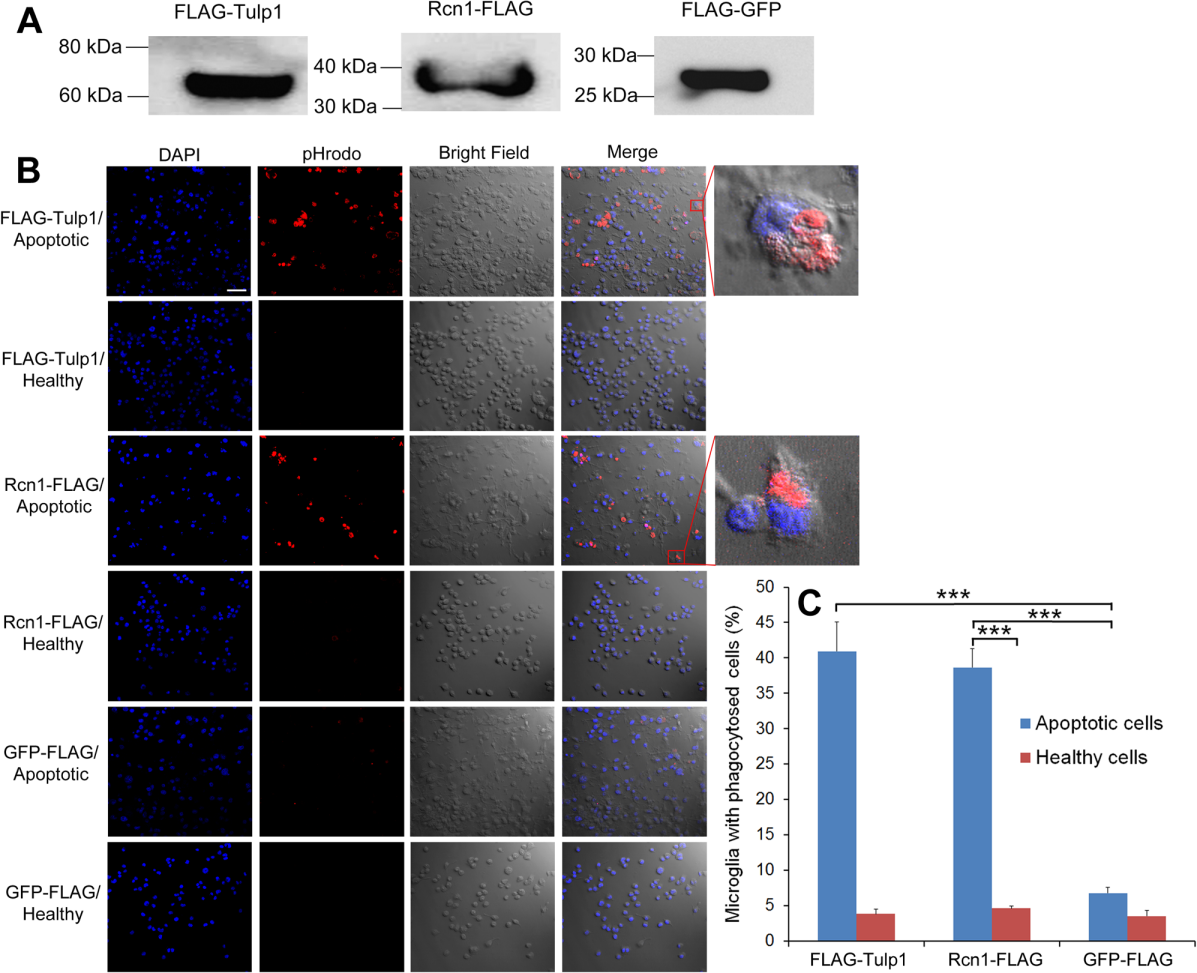
Rcn1 stimulates microglial phagocytosis. (A) Expression of Rcn1-FLAG, FLAG-Tulp1 and GFP-FLAG in Neuro-2A cells was verified by Western blot using anti-FLAG mAb. (B) Rcn1 facilitates BV-2 microglial phagocytosis. Rcn1-FLAG, FLAG-Tulp1 (positive control) or GFP-FLAG (negative control) was expressed in Neuro-2A cells. Cells were treated with or without etoposide to induce apoptosis, labeled with pHrodo, incubated with BV-2 microglia for phagocytosis, and analyzed by confocal microscopy. Scale bar = 50 μm. (C) Percentage of BV-2 cells with phagocytosed cargos in (B) were quantified by ImageJ (+ s.e.m., *** *P*<0.0001, n = 3, t-test).

To independently verify Rcn1 as a phagocytosis ligand, we expressed Rcn1-FLAG in Neuro-2A cells (Fig 2A), which were subsequently treated with or without etoposide to induce apoptosis. The apoptotic and healthy cells were labeled with pHrodo fluorogenic dye and incubated with BV-2 cells for phagocytosis. pHrodo is a pH-sensitive fluorogenic dye and is activated with increased fluorescence intensity in acidic phagosomes [17]. Coupled with confocal microscopy, pHrodo reliably distinguishes engulfed cargos. The results showed that Rcn1 was capable of stimulating microglial phagocytosis of apoptotic cells but not healthy cells (Fig 2B and 2C). Confocal z-stack images in high magnification are superimposed with the cognate bright fields to clearly indicate pHrodo signals inside microglia. FLAG-Tulp1 was previously characterized as a phagocytosis ligand for Mer receptor and included as a positive control. GFP-FLAG as a negative control failed to promote microglial phagocytosis of apoptotic cells.

**Fig 3:**
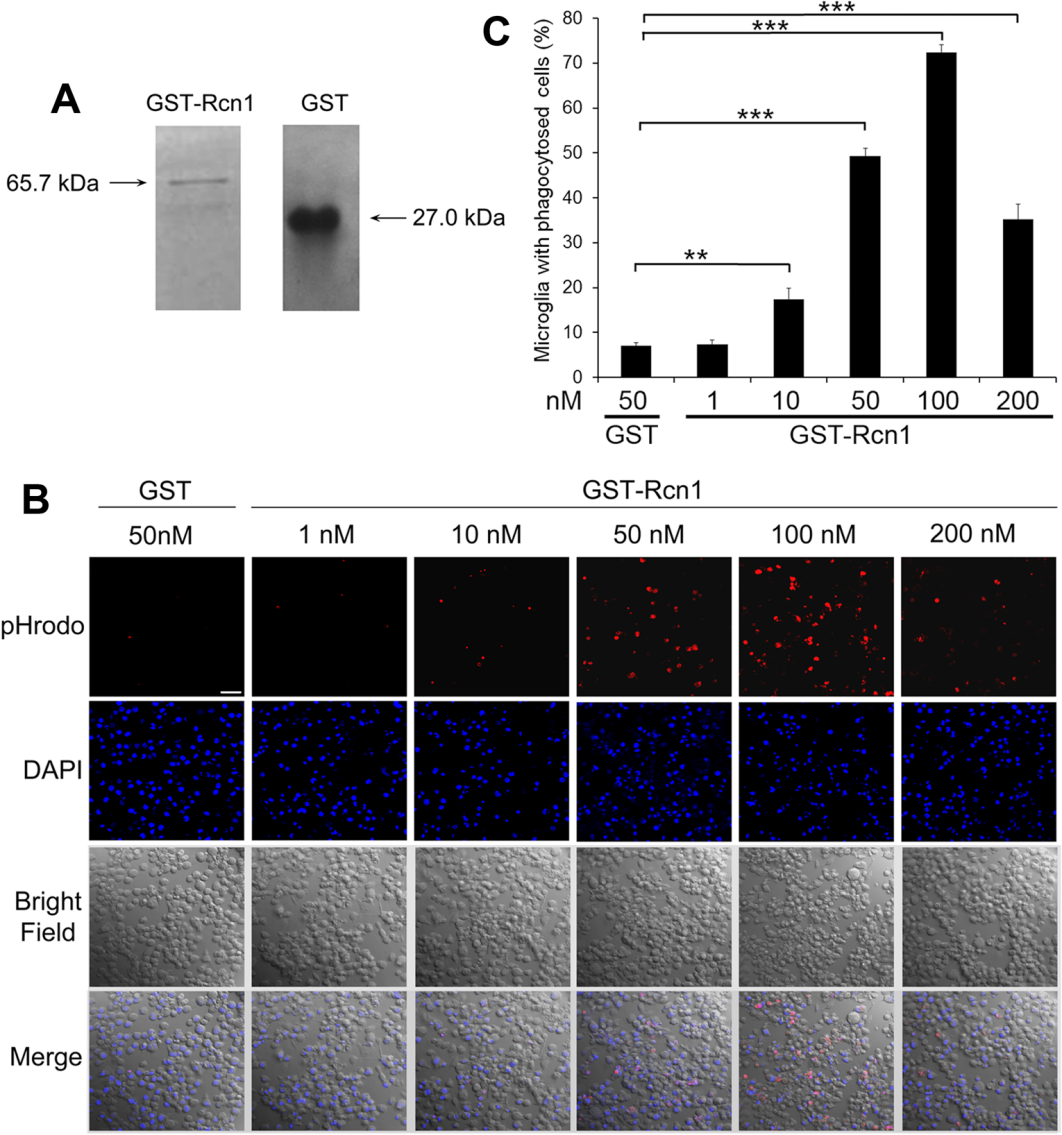
Purified Rcn1 stimulates microglial phagocytosis. (A) Purification of GST-Rcn1 and GST. Both proteins were expressed in bacteria, purified with glutathione columns and analyzed by SDS-PAGE. (B) Rcn1-facilitates microglial phagocytosis. Neuro-2A cells were treated with or without etoposide to induce apoptosis, labeled with pHrodo, and incubated with BV-2 microglia in the presence of the purified protein at indicated concentrations. Phagocytosed pHrodo signals were analyzed by confocal microscopy. Scale bar = 50 μm. (C) Percentage of BV-2 cells with phagocytosed cargos in (B) were quantified (+ s.e.m., *** *P*<0.0001, n = 3, t-test).

We verified Rcn1 stimulation of microglial phagocytosis with purified GST-Rcn1 fusion protein (Fig 3A). The results showed that GST-Rcn1 facilitated microglial engulfment of apoptotic cells in a dose-dependent manner (Fig 3B and 3C). GST alone minimally induced phagocytosis. GST-Rcn1 (100 nM) resulted in 72.4% of microglia with engulfed apoptotic cells. However, GST-Rcn1 at 200 nM had a reduced capacity to promote microglial phagocytosis, a phenomenon discussed in previous studies for soluble bridging molecules as a subtype of phagocytosis ligands [17,21]. These results suggest that Rcn1 is a microglial phagocytosis ligand.

To eliminate the possibility that Rcn1 may enhance general phagocytic activity of microglia to engulf all types of cargos non-specifically, we performed microglial phagocytosis with fluorescent latex beads in the presence of GST-Rcn1 or GST control. The results revealed that Rcn1 failed to facilitate microglial phagocytosis of latex beads (S2 Fig), suggesting that Rcn1 specifically recognizes apoptotic ells and facilitates their clearance.

### Rcn1 extracellular trafficking and selective binding to apoptotic cells

**Fig 4:**
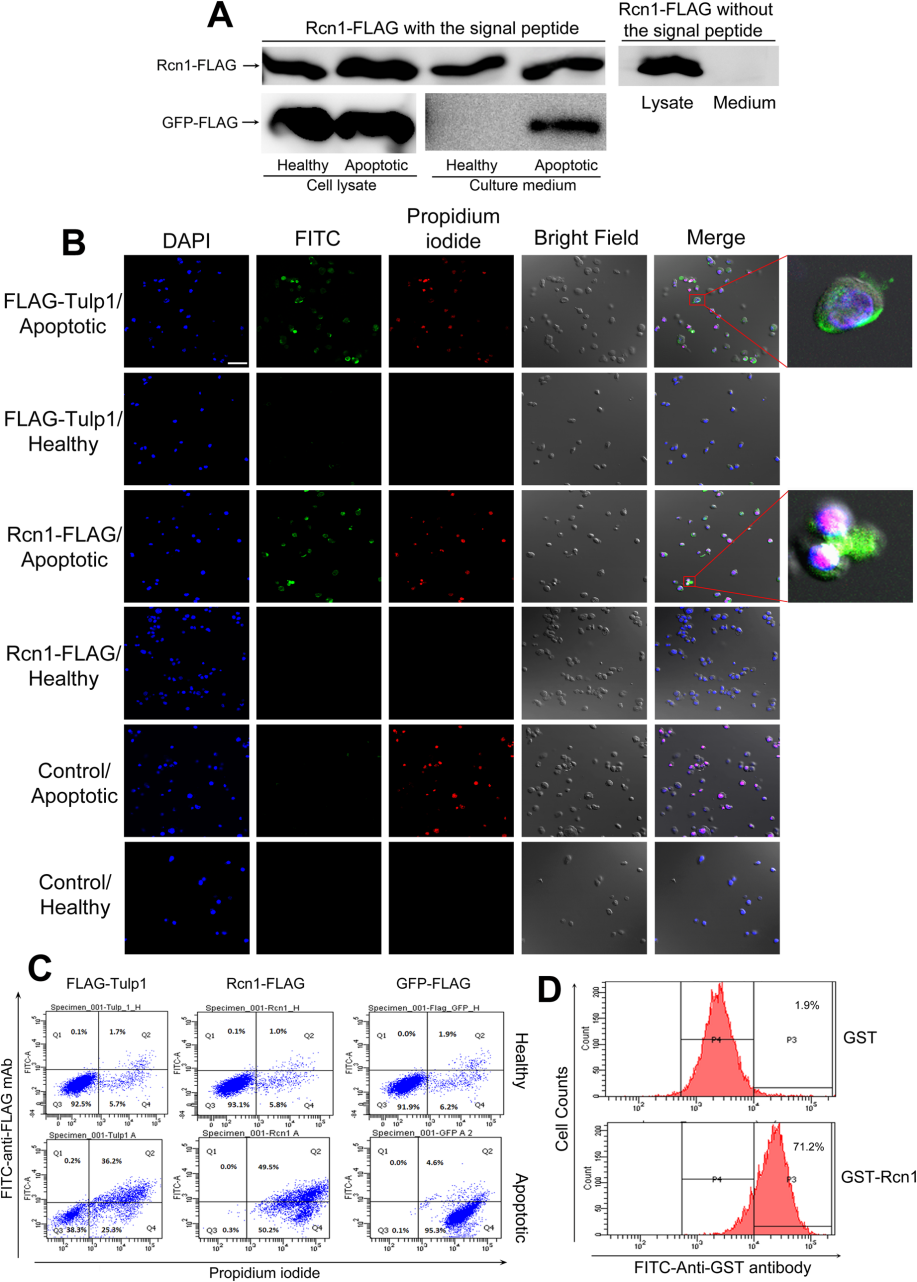
Rcn1 selectively binds to the surface of apoptotic neurons but not healthy neurons. (A) Rcn1 is a secreted protein. Rcn1-FLAG with or without the signal peptide was expressed in Neuro-2A cells. Rcn1-FLAG in the conditioned medium of the apoptotic or healthy cells was immunoprecipitated with anti-FLAG mAb and analyzed by Western blot. GFP-FLAG was a non-secretory protein control. (B) Rcn1 selectively binds to apoptotic cells. Rcn1-FLAG was expressed in Neuro-2A cells. Cell surface-bound Rcn1-FLAG was detected for apoptotic and healthy Neuro-2A cells using FITC-labeled anti-FLAG mAb and analyzed by confocal microscopy. FLAG-Tulp1 is a positive control. Apoptotic cells were labeled with propidium iodide. Scale bar = 50 μm. (C) Flow cytometry analysis of Rcn1 binding to apoptotic neurons. Lysates were prepared from cells expressing Rcn1-FLAG, FLAG-Tulp1 (positive control) or GFP-FLAG (negative control), incubated with apoptotic or healthy Neuro-2A cells and analyzed by flow cytometry using FITC anti-FLAG mAb. (D) Rcn1 binds to microglia surface. GST-Rcn1 or GST control was incubated with BV-2 cells and analyzed by flow cytometry using FITC-anti-GST antibody.

One of the criteria for a protein to be qualified as a phagocytosis ligand is its extracellular trafficking for access to phagocytosis cargos and receptors. Phagocytosis ligands can be either secreted through conventional or nonconventional pathways, or passively released from apoptotic cells [19]. Rcn1 with a classical signal peptide and an ER retention signal [10] was originally reported to be strictly localized in the ER [22]. A recent study indicated that Rcn1 also presents on the surface of bone endothelial cells and prostate cancer cells [23]. However, soluble extracellular Rcn1 has not been reported. We analyzed Rcn1 in the conditioned culture medium of apoptotic and healthy Neuro-2A cells expressing Rcn1-FLAG by immunoprecipitation with anti-FLAG mAb. The results showed that Rcn1 was detected in the conditioned medium of both apoptotic and healthy cells (Fig 4A), suggesting that Rcn1 can be secreted as a soluble protein. Rcn1-FLAG without the signal peptide failed to be detected in the conditioned medium, suggesting that Rcn1 is a secreted protein. GFP-FLAG as a non-secretory protein control was released only from apoptotic cells but not healthy cells.

When BV-2 cells were pre-transfected with control or Rcn1-FLAG plasmid containing the signal peptide, only Rcn1-FLAG-expressing BV-2 cells showed an increase in phagocytosis of apoptotic cells (S1 Fig). Together with Fig 2, these results suggest that Rcn1 can be released from either neurons or microglia to facilitate microglial phagocytosis of apoptotic cells.

### Rcn1 selectively binds to apoptotic but not healthy cells

Another criterion of phagocytosis ligands is that they should discriminatively bind to apoptotic cells or other intended phagocytosis cargos, but not healthy cells, so that microglia will not phagocytose and destroy healthy neurons alive [24]. To test if Rcn1 meets this criterion, we analyzed its binding to apoptotic and healthy cells. Rcn1-FLAG-expressing Neuro-2A cells were treated with or without etoposide to induce apoptosis. Cell surface-bound Rcn1 was detected by FITC-labeled anti-FLAG mAb and analyzed by confocal microscopy. The results showed that Rcn1 only bound to apoptotic neurons but not healthy neurons (Fig 4B). FLAG-Tulp1 as a positive control had similar preferential binding to apoptotic Neuro-2A cells.

Furthermore, we characterized Rcn1 binding to apoptotic neurons by flow cytometry. We expressed Rcn1-FLAG in HEK293T cells and prepared the cell lysate. Neuro-2A cells were induced apoptosis with or without etoposide and incubated with the cell lysate, followed by FITC anti-FLAG mAb. Flow cytometry analysis revealed that Rcn1-FLAG bound only to apoptotic cells but not healthy cells (Fig 4C). FLAG-Tulp1 had a similar binding pattern. GFP-FLAG bound to neither apoptotic nor healthy cells. Thus, both confocal microscopy and flow cytometry analyses showed the consistent results that Rcn1 selectively bound to apoptotic but not healthy neurons, suggesting that Rcn1 meets the criterion as a phagocytosis ligand.

Phagocytosis ligands should bind to their cognate receptors on phagocyte surface. Our flow cytometry analysis indicated that GST-Rcn1 but not GST bound to BV-2 cells (Fig 4D), suggesting that Rcn1 can recognize its unknown receptor on microglia.

### Rcn1 targets ingested cargos to phagosomes

**Fig 5:**
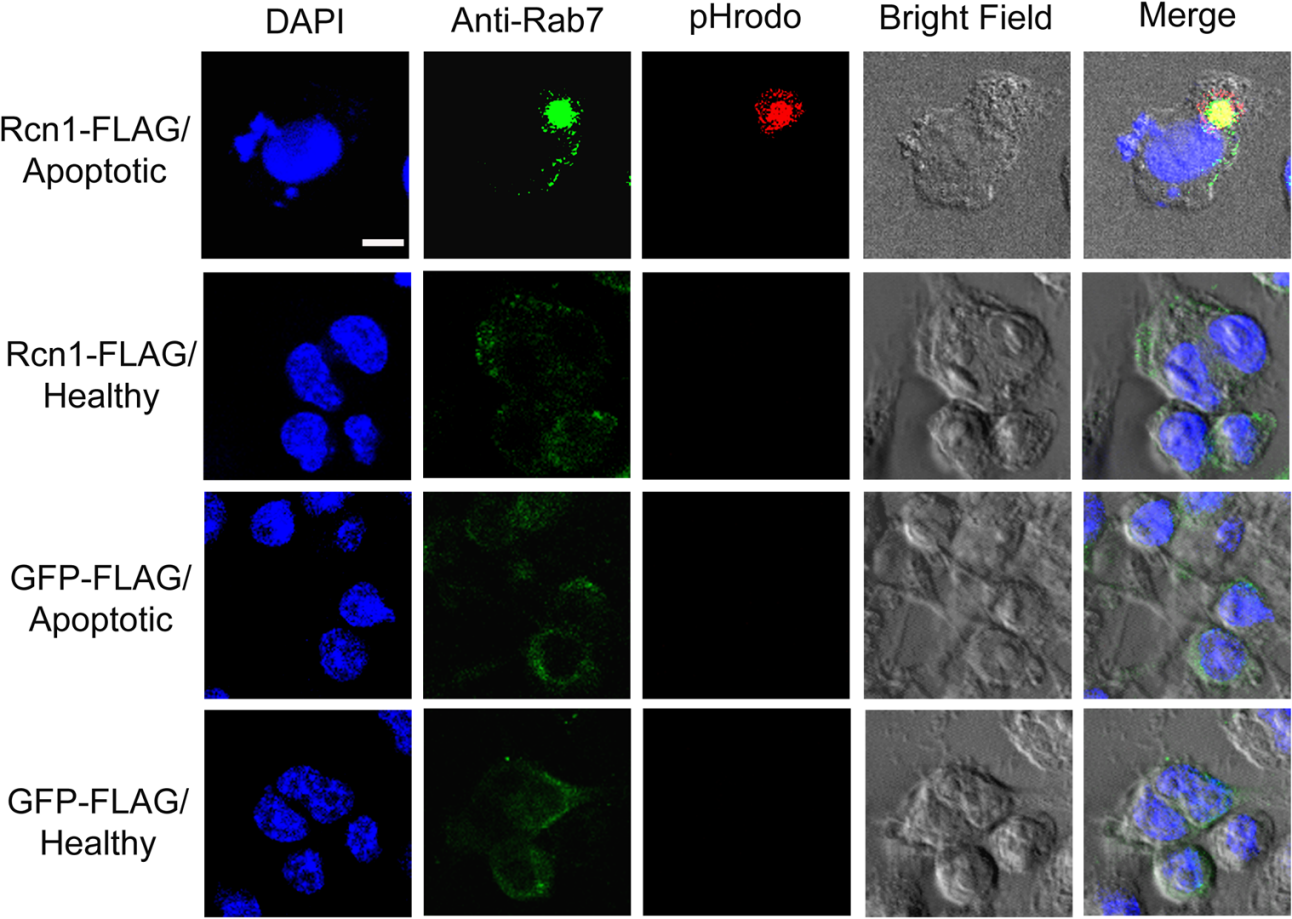
Rcn1 mediates microglial engulfment via phagocytosis pathway. Phagocytosis of apoptotic cells by BV-2 microglia was performed as in Fig 2A. Phagosome marker Rab7 was detected using anti-Rab7 antibody and FITC-labeled secondary antibody, and analyzed by confocal microscopy. The z-stack images of pHrodo and FITC are co-localized and superimposed with cognate DAPI signals and bright fields. Bar = 10 μm.

We verified Rcn1-mediated engulfment of pHrodo-labeled apoptotic cells via microglial phagocytosis pathway by analyzing the distribution of phagosome biomarker Rab7. Immunocytochemistry showed that Rab7 was colocalized with ingested pHrodo signals (Fig 5), suggesting that Rcn1-mediated engulfment targeted the apoptotic cells to phagosomes.

### Rcn1 facilitates macrophage phagocytosis

**Fig 6:**
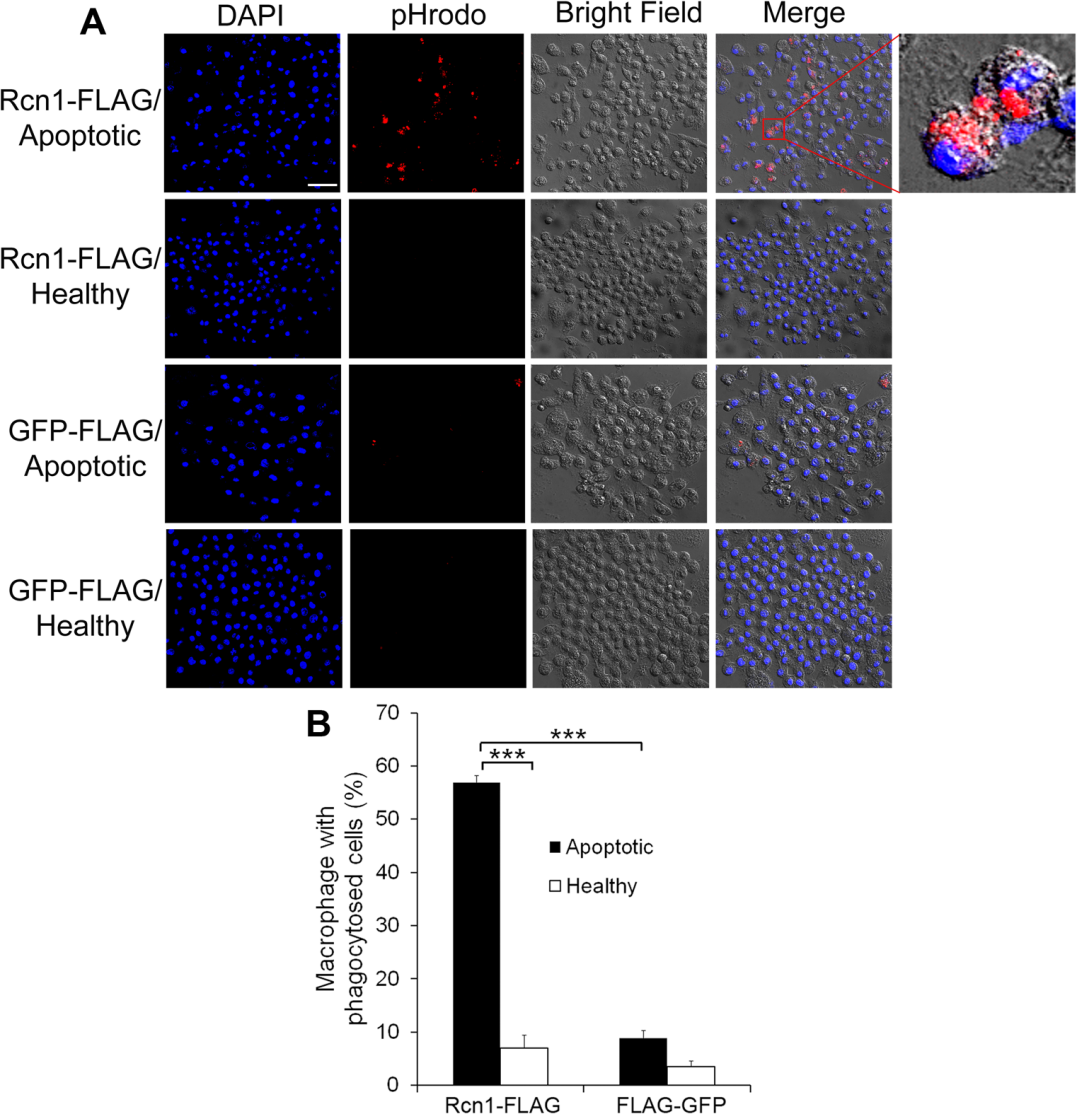
Rcn1 facilitates macrophage phagocytosis. (A) Rcn1 stimulates macrophage phagocytosis of apoptotic neurons. Healthy or apoptotic Neuro-2A cells were labeled with pHrodo, incubated with J774 macrophage cells for phagocytosis and analyzed by confocal microscopy as in Fig 2A. The z-stack images of pHrodo and DAPI are superimposed with the cognate bright fields to reveal phagocytosed cargos. (B) Percentage of macrophages with phagocytosed cargos in (A) were quantified. Bar = 50 μm (+ s.e.m., *** *P*<0.0001, n = 3, t-test).

Macrophages and microglia share many similarities in functional roles and molecular regulations. We characterized Rcn1 capacity to promote macrophage phagocytosis of apoptotic cells. The results showed that Rcn1 stimulated phagocytosis of apoptotic cells, but not healthy cells, by J774 macrophages (Fig 6), suggesting that Rcn1 is a phagocytosis ligand for both microglia and macrophages.

## Discussion

### Rcn1 as a microglial phagocytosis ligand

Rcn1 is the first protein identified with EF-hand motifs and is predominantly expressed in the ER with an ER retention signal of HDEL [10,11,22]. Rcn1 was recently reported to be detected on the surface of bone endothelial cells and prostate cancer cells [23]. The surface expression on the endothelial cells is upregulated by tumor necrosis factor-α (TNF-α). Our results showed that Rcn1 can be secreted into the extracellular environment (Fig 4A) and preferentially bind to apoptotic but not healthy neurons. We speculate that TNF- α-induced apoptosis may be responsible for the upregulation of Rcn1 on cell surface [23] due to its preferential binding to apoptotic cells. Rcn1 expression is upregulated in limb-girdle muscular dystrophy [25] as well as a number of cancerous cell lines, such as invasive breast cancer [26] and colorectal cancer [27].

Deletion of a large genomic region surrounding Rcn1 is lethal in early stage of the homozygotic mice, implicating its importance in development [28]. However, its functional roles are poorly defined. A recent screening study with mammalian two-hybrid system identified Rcn1 as a MEK1-binding protein [29]. Overexpression or silencing studies indicated that Rcn1 negatively regulated phenylephrine-induced cardiomyocyte hypertrophy [29]. However, the physiological access of Rcn1 in the ER and extracellular space to cytoplasmic MEK1 remains to be demonstrated. It cannot be eliminated that Rcn1 may negatively regulate the hypertrophy via ER process of other cardiomyocyte regulatory proteins. Sec63p is an ER protein identified as an Rcn1-binding partner with unknown functional roles [30].

This study identified and independently characterized Rcn1 as a microglial phagocytosis ligand with multiple lines of evidence, including its preferential binding to apoptotic neurons and stimulation of microglial phagocytosis. Moreover, Rcn1-mediated phagocytosis targets the ingested cargos to microglial phagosomes. Endothelial cells are well-known non-professional phagocytes [31,32]. Thus, Rcn1 on endothelial cell surface [23] may facilitate phagocytosis of serological debris or deposits on the cell surface to prevent debris buildup and atherosclerosis.

Gas6 and Tulp1 are known phagocytosis ligands for Mer receptor [18,20] and were identified as the most active ligands in Table 1 by OPD-NGS. We wonder if Rcn1 may also bind to Mer receptor on microglia. Co-immunoprecipitation showed no interaction between Rcn1-FLAG and Mer-Fc (data not shown), even though this assay successfully revealed the interactions of tubby and Tulp1 with Mer receptor [18]. As a result, the identity of Rcn1 receptor remains elusive. Thus far, we only know that this receptor is expressed on the surface of both microglia and endothelial cells [23]. Identification of Rcn1 receptor will help understand its functional roles and molecular mechanisms.

### Phagocytosis ligands are the key to defining the functional roles of microglial phagocytosis

During neurogenesis, excess immature neurons are generated, deleted via apoptosis and removed by microglial phagocytosis [6,33]. Synaptic remodeling occurs throughout our life, and microglial phagocytosis participates in the removal of old synaptic material [34]. Although microglia may contribute to the initiation of multiple sclerosis via antigen phagocytosis and presentation, microglial phagocytosis of damaged cells and myelin debris in the recovery phase is considered beneficial to the resolution of the autoimmune disease and neuronal regeneration [35]. Infiltrated macrophages and resident microglia are critical to the clearance of myelin debris during traumatic brain injury [6,36]. Microglial clearance of Aβ, including antibody-mediated phagocytosis, prevents amyloid plaques and Alzheimer’s disease [37,38]. These knowledge indicates that microglial phagocytosis plays an important role in maintaining neuronal homeostasis. However, the challenge is not only what cargos microglia clear but also how these cargos are recognized by microglia.

Phagocytosis ligands are the key to cargo recognition and consist of “eat-me” signals and soluble bridging molecules [39]. The former are anchored on cargo surface and bind to phagocytic receptors either directly or indirectly via bridging molecules. Ligand-cargo recognition directly defines the functional roles of phagocytes, including microglia. Phagocytosis ligands can be either proteins (e.g., calreticulin) or non-protein molecules (e.g., externalized phosphatidylserine) on the surface of apoptotic cells or other cargos [1,39,40]. Like other cellular ligands, however, phagocytosis ligands are traditionally identified on individual cases with daunting technical challenges.

OPD/PFC was recently developed to tackle the technical challenges [15,19]. The main distinction between OPD and conventional phage display is that OPD can identify real endogenous ligands, as opposed to out-of-frame unnatural peptides [41,42]. Using this approach, we successfully identified and independently validated Tulp1 as a genuine phagocytosis ligand [15,18]. Two other proteins, Lyar and ABCF1, were also identified [15] and independently verified as RPE phagocytosis ligands (unpublished data, Guo F., et al.). These results suggest that OPD/PFC is a valid approach for unbiased identification of phagocytosis ligands. In fact, phagocytosis ligands are the only signaling molecules that can be identified in an unbiased manner by OPD/PFC in the absence of receptor information [15,19]. Identified ligands can be used as molecular probes to further delineate cognate eat-me signals or phagocytic receptors [18].

However, manual screening of individual clones enriched by OPD/PFC in our previous studies was a time-consuming and labor-intensive procedure to identify unknown ligands [15]. The combination of OPD with NGS in this study dramatically improved the efficiency to identify unknown phagocytosis ligands. Independent characterization of Rcn1 in this study supports the validity of OPD-NGS as a new functional screening approach for systematic identification of phagocytosis ligands.

Additional advantage of OPD-NGS is possible functional activity quantification. The copy numbers of cDNA inserts identified by NGS may reflect the relative abundance of enriched clones or internalization activity of cognate ligands. We are now investigating the reliability of OPD-NGS for activity quantification of microglial ligand profile. This will enable future activity comparison to systematically identify disease- or age-related ligands for microglial phagocytosis. Such comparison will also efficiently map the similarities and differences between microglia and macrophages.

## Conclusions

**Fig 7:**
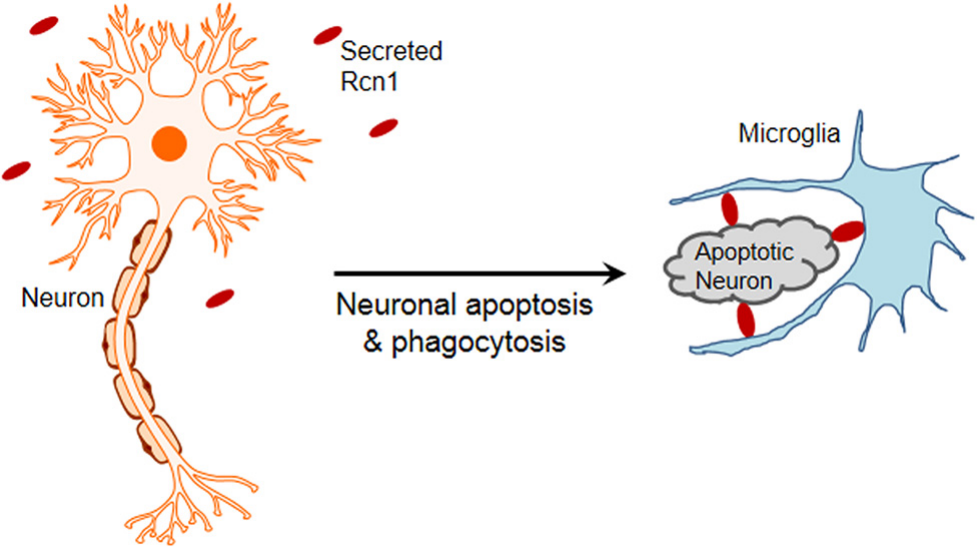
A cartoon model for Rcn1 to facilitate microglial phagocytosis. Rcn1 is secreted from neurons or other cells and preferentially binds to apoptotic neurons as a bridging molecule to facilitate their clearance by microglial phagocytosis.

This study identified and characterized Rcn1 as a novel microglial phagocytosis ligand (Fig 7). The new functional screening approach described in this study is the only available method for systematic identification of microglial phagocytosis ligands in the absence of receptor information. This method should be broadly applicable to many other phagocytes to unravel the mystery of molecular phagocyte biology.

## Supporting Information

S1 Fig Rcn1 expression in BV-2 cells facilitates microglial phagocytosis of apoptotic cells. (A) BV-2 cells were transfected with Rcn1-FLAG or control plasmid for 48 h and analyzed for the expression of Rcn1-FLAG by Western blot using anti-FLAG mAb (50 μg protein/lane). (B) Rcn1-expressing or control BV-2 cells were incubated with pHrodo-labeled apoptotic or healthy Neuor-2A cells for phagocytosis. Engulfed cells were analyzed, as described in Fig 2B. Bar = 50 μm. (C) Percentage of BV-2 cells with phagocytosed cargos in (B) were quantified by ImageJ (+ s.e.m., n = 3, t-test).
(PDF)

S2 Fig Rcn1 does not enhance phagocytosis of latex beads by microglia. BV-2 cells were incubated with FITC-labeled latex beads in the presence of GST-Rcn1 or GST control (100 nM) for phagocytosis, as described in Fig 2B. Bar = 50 μm. (B) Percentage of BV-2 cells with phagocytosed cargos in (A) were quantified by ImageJ (+ s.e.m., n = 3, t-test).
(PDF)
