# Supplementary material for: Reticulocalbin-1 Facilitates Microglial Phagocytosis
Source: PLoS One. 2015 May 18;10(5):e0126993. doi: 10.1371/journal.pone.0126993 (PMC4436338; doi:10.1371/journal.pone.0126993)
Supplement: S1 Fig — (A) BV-2 cells were transfected with Rcn1-FLAG or control plasmid for 48 h and analyzed for the expression of Rcn1-FLAG by Western blot using anti-FLAG mAb (50 μg protein/lane). (B) Rcn1-expressing or control BV-2 cells were incubated with pHrodo-labeled apoptotic or healthy Neuor-2A cells for phagocytosis. Engulfed cells were analyzed, as described in Fig 2B. Bar = 50 μm. (C) Percentage of BV-2 cells with phagocytosed cargos in (B) were quantified by ImageJ (+ s.e.m., n = 3, t-test). (PDF) [file pone.0126993.s001.pdf]

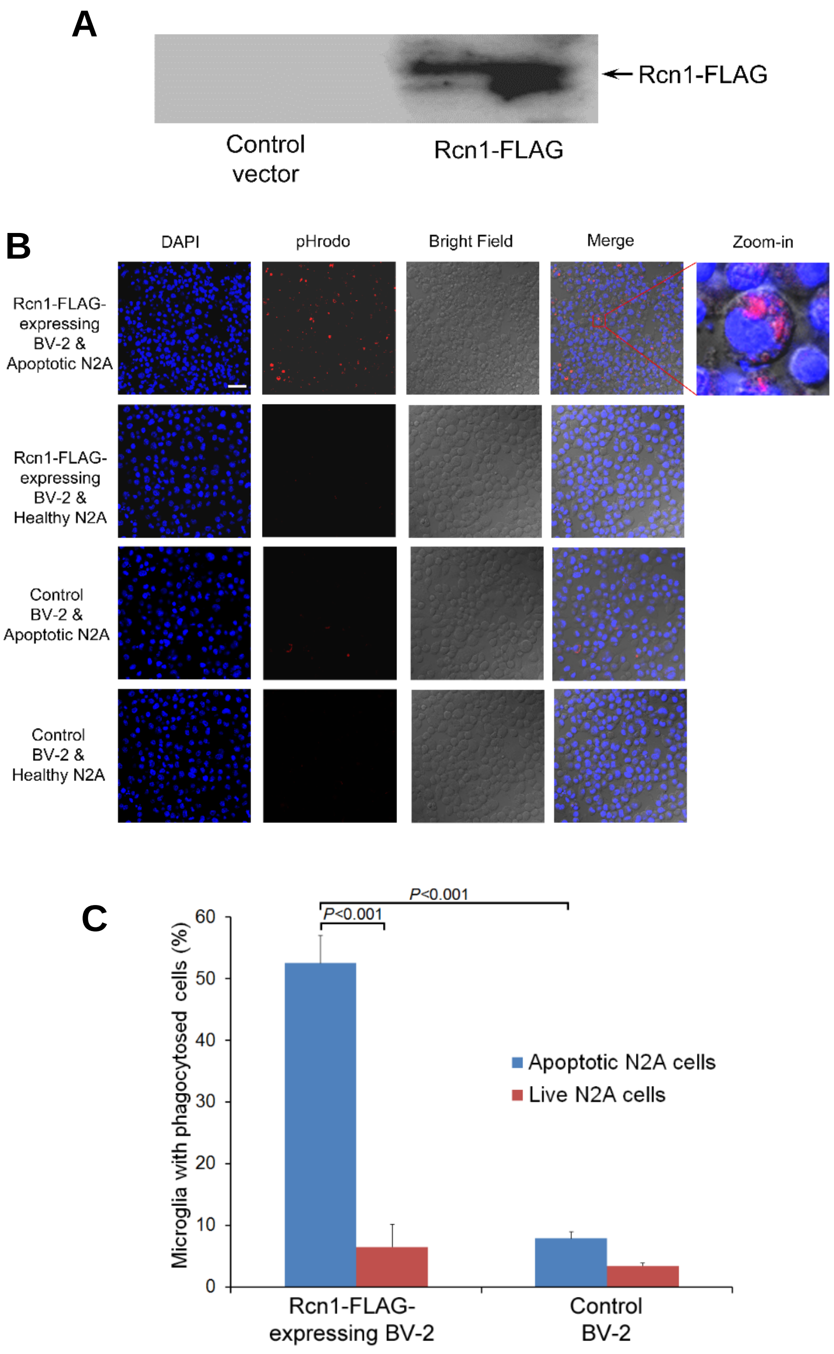

S1 Fig. **Rcn1 expression in BV-2 cells facilitates microglial phagocytosis of apoptotic cells**. (A) BV-2 cells were transfected with Rcn1-FLAG or control plasmid for 48 h and analyzed for the expression of Rcn1-FLAG by Western blot using anti-FLAG mAb (50 µg protein/lane). (B) Rcn1-expressing or control BV-2 cells were incubated with pHrodo-labeled apoptotic or healthy Neuor-2A cells for phagocytosis. Engulfed cells were analyzed, as described in Fig. 2B. Bar = 50 µm. (C) Percentage of BV-2 cells with phagocytosed cargos in (B) were quantified by ImageJ (± s.e.m., n=3, t-test).

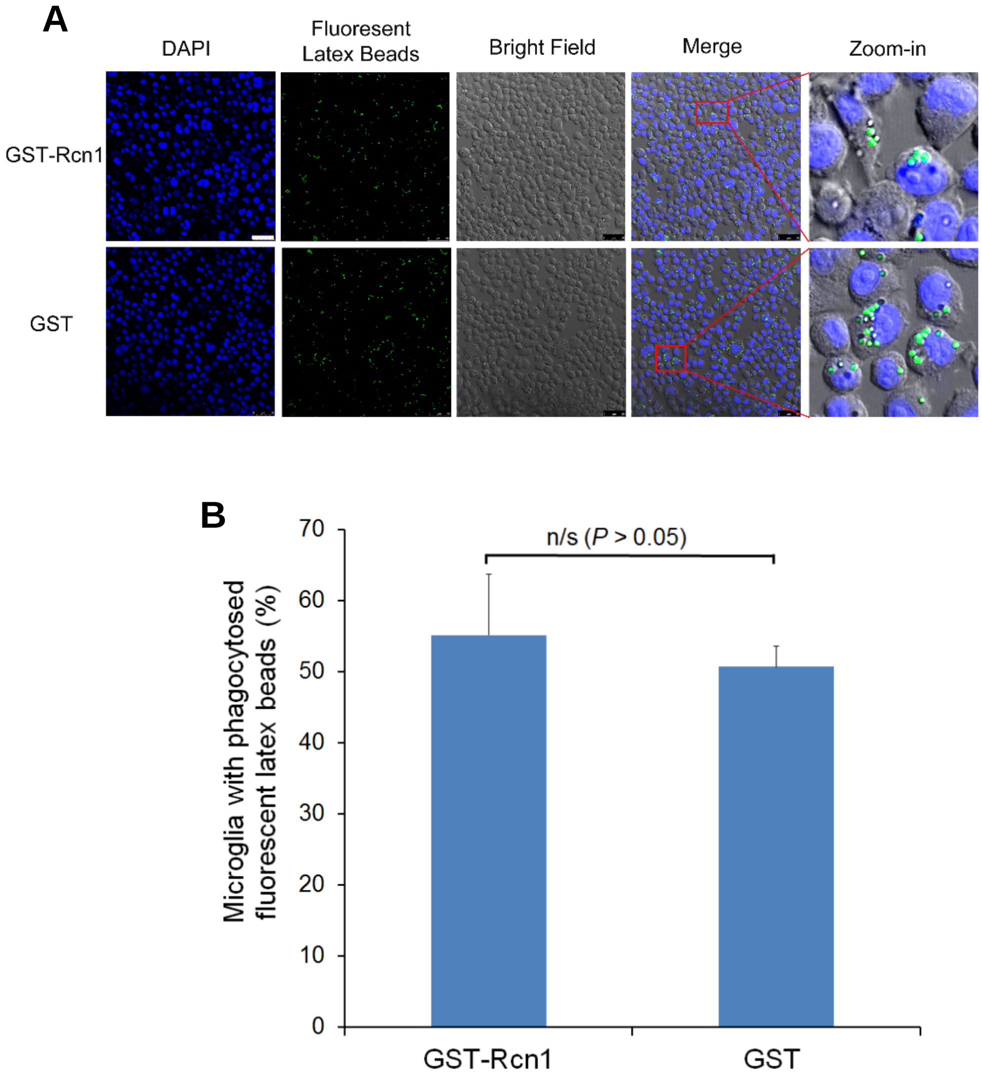

S2 Fig. Rcn1 does not enhance phagocytosis of latex beads by microglia. BV-2 cells were incubated with FITC-labeled latex beads in the presence of GST-Rcn1 or GST control (100 nM) for phagocytosis, as described in Fig. 2B. Bar = 50 μm. (B) Percentage of BV-2 cells with phagocytosed cargos in (A) were quantified by ImageJ (± s.e.m., n=3, t-test).
