# Supplementary material for: Reticulocalbin-1 Facilitates Microglial Phagocytosis
Source: PLoS One. 2015 May 18;10(5):e0126993. doi: 10.1371/journal.pone.0126993 (PMC4436338; doi:10.1371/journal.pone.0126993)
Supplement: S2 Fig — BV-2 cells were incubated with FITC-labeled latex beads in the presence of GST-Rcn1 or GST control (100 nM) for phagocytosis, as described in Fig 2B. Bar = 50 μm. (B) Percentage of BV-2 cells with phagocytosed cargos in (A) were quantified by ImageJ (+ s.e.m., n = 3, t-test). (PDF) [file pone.0126993.s002.pdf]

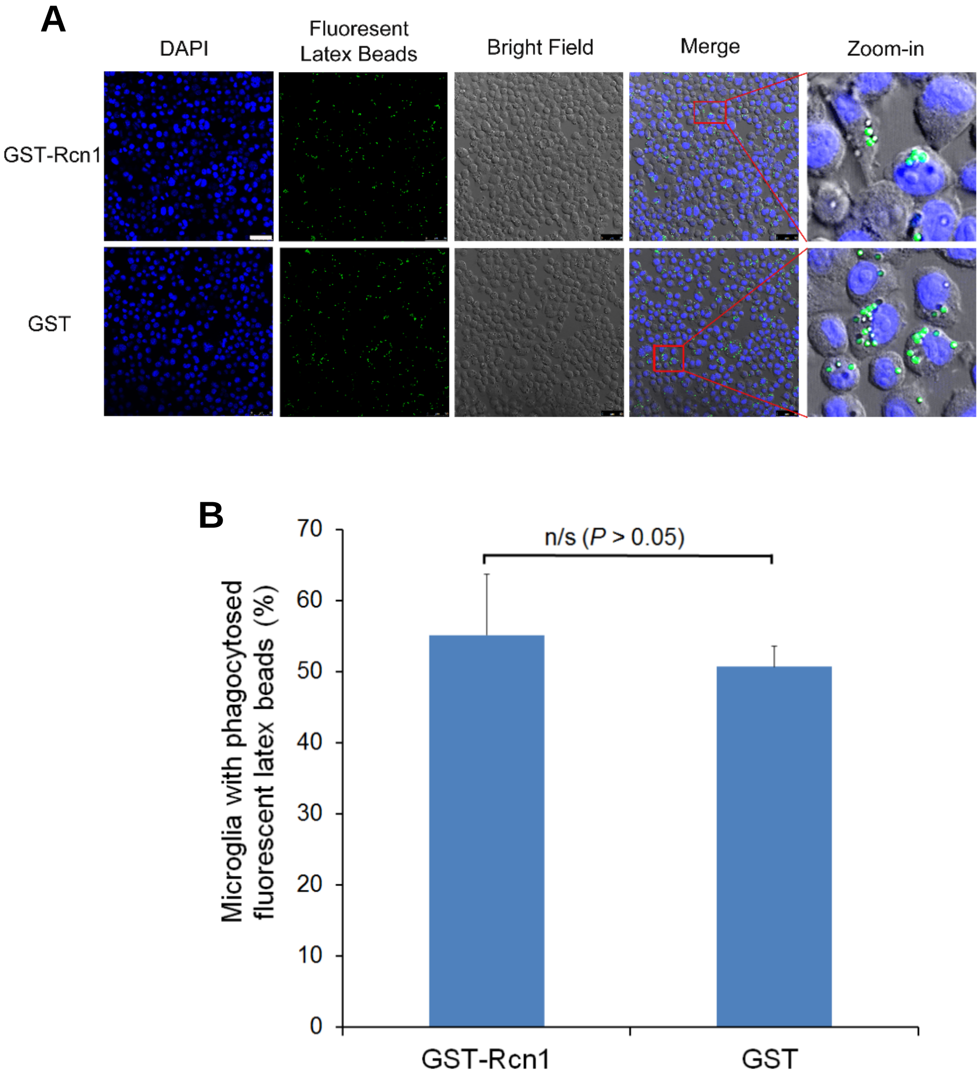

S2 Fig. Rcn1 does not enhance phagocytosis of latex beads by microglia. BV-2 cells were incubated with FITC-labeled latex beads in the presence of GST-Rcn1 or GST control (100 nM) for phagocytosis, as described in Fig. 2B. Bar = 50 µm. (B) Percentage of BV-2 cells with phagocytosed cargos in (A) were quantified by ImageJ (± s.e.m., n=3, t-test).
